# C3G down-regulation enhances pro-migratory and stemness properties of oval cells by promoting an epithelial-mesenchymal-like process

**DOI:** 10.7150/ijbs.73192

**Published:** 2022-09-25

**Authors:** Nerea Palao, Celia Sequera, Ángel M Cuesta, Cristina Baquero, Paloma Bragado, Alvaro Gutierrez-Uzquiza, Aránzazu Sánchez, Carmen Guerrero, Almudena Porras

**Affiliations:** 1 Departamento de Bioquímica y Biología Molecular, Facultad de Farmacia, Universidad Complutense de Madrid; 28040 Madrid, Spain.; 2 Instituto de Investigación Sanitaria del Hospital Clínico San Carlos (IdISSC), 28040 Madrid, Spain.; 3 Aix-Marseille Univ, CNRS, Developmental Biology Institute of Marseille (IBDM), Turing Center for Living Systems, Parc Scientifique de Luminy, 13009 Marseille, France.; 4 Instituto de Biología Molecular y Celular del Cáncer (IBMCC), Universidad de Salamanca-CSIC, 37007 Salamanca, Spain.; 5 Instituto de Investigación Biomédica de Salamanca (IBSAL), 37007 Salamanca, Spain.; 6 Departamento de Medicina, Universidad de Salamanca, 37007 Salamanca, Spain.

**Keywords:** C3G, oval cells, migration, epithelial mesenchymal transition, stemness

## Abstract

Previous data indicate that C3G (RapGEF1) main isoform is highly expressed in liver progenitor cells (or oval cells) compared to adult mature hepatocytes, suggesting it may play an important role in oval cell biology. Hence, we have explored C3G function in the regulation of oval cell properties by permanent gene silencing using shRNAs. We found that C3G knock-down enhanced migratory and invasive ability of oval cells by promoting a partial epithelial to mesenchymal transition (EMT). This is likely mediated by upregulation of mRNA expression of the EMT-inducing transcription factors, *Snail1*, *Zeb1* and *Zeb2*, induced in C3G-silenced oval cells. This EMT is associated to a higher expression of the stemness markers, CD133 and CD44. Moreover, C3G down-regulation increased oval cells clonogenic capacity by enhancing cell scattering. However, C3G knock-down did not impair oval cell differentiation into hepatocyte lineage. Mechanistic studies revealed that HGF/MET signaling and its pro-invasive activity was impaired in oval cells with low levels of C3G, while TGF-β signaling was increased. Altogether, these data suggest that C3G might be tightly regulated to ensure liver repair in chronic liver diseases such as non-alcoholic steatohepatitis. Hence, reduced C3G levels could facilitate oval cell expansion, after the proliferation peak, by enhancing migration.

## Introduction

Adult hepatic progenitor cells (HPCs), also known as oval cells in rodents, are bipotent cells that expand after chronic liver damage and can differentiate into hepatocytes or cholangiocytes to repair liver when hepatocytes are unable to do it 1, 2. Thus, during severe and chronic liver damage, HPCs expand, contributing to liver repair. Due to this function and its potentiality in therapy, full understanding of oval cell biology and how they are regulated is an important issue, considering that oval cells could have a potential implication in the generation of liver fibrogenesis and/or development of hepatocarcinoma (HCC) 2, 3.

Different signals are involved in the regulation of oval cells. Among them, some signals generated by hepatic stellate cells (HSCs)/myofibroblasts, which are important components of oval cells niche, are key players, including HGF, TGF-β, TGF-α, EGF and b-FGF. Additional important signals are TWEAK, IL-6 or TNF-α, secreted by inflammatory cells 3. It is worth highlighting the relevance of HGF, which has broad-ranging regulatory activities, inducing oval cell survival, migration, invasion, and differentiation, critically counterbalancing TGF-β actions 4-8, being required for the repopulation capacity of oval cells in the injured liver 9. Nevertheless, the regeneration process mediated by HPCs and its regulation has not been fully characterized yet.

C3G (Crk SH3-domain-binding guanine-nucleotide-releasing factor) protein is a guanine-nucleotide exchange factor (GEF) for Rap1 and other Ras proteins 10. However, C3G can also act through mechanisms independent of its GEF activity 11, 12, likely mediated by its interaction with other proteins through its proline-rich domain and/or its translocation to the nucleus 13. C3G is a protein ubiquitously expressed, although some tissue-specific differences exist 10, 14. The main isoform is a protein with an apparent molecular weight of 140 kDa, which constitutes the isoform A. Other common isoforms are the B one, which differs in 21 aminoacids (aa) from the N-terminal region, and a mouse isoform with a deletion of 38aa in the N-terminal region 10. A p87C3G isoform has also been described in chronic leukemia cells 15 and more recently, a new one of 175kDa was identified in the brain 16. C3G is required for embryonic development 17 and regulates several cellular functions such as adhesion, migration, apoptosis, and differentiation 18-21.

C3G is expressed in mouse embryonic liver, where an enrichment in shorter isoforms, as compared to brain, was described 22. We also found that C3G main isoform is expressed in neonatal and adult hepatocytes, as well as in oval cells, but at different levels 23, 24. Thus, C3G protein levels are high in both oval cells and neonatal hepatocytes, but low in adult hepatocytes. Moreover, C3G is upregulated in HCC cells, as compared to adult hepatocytes, promoting tumor growth 23. Therefore, our data show that C3G levels are tightly regulated in the liver during development and their dysregulation has pathological implications, suggesting it might play a key role in oval cell function. In this work, we have evaluated the role of C3G in the regulation of oval cell biology using a gene silencing approach.

## Materials and methods

### Cell culture, C3G silencing and treatments

Oval cell lines (wt) were previously generated from met^flx/flx^ mice maintained on a 0,1% 3,5-diethoxycarbonyl-1,4-dihydro-collidine (DDC)-supplemented diet for 13 days to get an expansion of oval cells as described 5. Permanent C3G silencing was performed using Lentiviral Particles (75,000 infectious units) containing a home-designed shRNA (shC3G-1): forward (5´-3´) GATCCCCGCCCTCTCCTCCTGTTATATTCAAGAGATATAACAGGAGGAGAGGGCTTTTTGGAAA and reverse (5´-3´) AGCTTTTCCAAAAAGCCCTCTCCTCCTGTTATATCTCTTGAATATAACAGGAGGAGAGGGCGGG or a mixture of different shRNAs (SCBT, sc-29864-V) (shC3G-3) in the presence of polybrene (10 μg/mL SCBT, sc-134220). A control shRNA was used for non-silenced cells. Cells were selected by either GFP expression (shC3G-1) or puromycin (2 µg/mL, Panreac#A2856) (shC3G-3). A pool of silenced cells was used for the experiments.

Cells were maintained in DMEM supplemented with 10% fetal bovine serum (FBS) at 37ºC and 5% CO_2_. Cells were serum starved for 2-12 h prior to stimulation with HGF (20 ng/mL) or TGF-β (1 ng/ml).

### Wound healing and invasion assays

Wound healing was performed as described 21, 25. Invasion was assayed in Matrigel (333 µg/cm2; Corning#356234) coated transwells (BD#353097). 50,000 cells were seeded in the upper chamber in serum-free medium. 10% FBS-medium, placed in the lower chamber, acted as chemoattractant. After 24h, cells from the lower chamber were fixed with 4% paraformaldehyde (PFA), stained with crystal violet and counted using an Eclipse TE300 Nikon microscope. To evaluate HGF effect on invasion, cells in the upper chamber were treated with this growth factor (20 ng/mL) and no serum was added into the lower chamber.

### Adhesion and clonogenicity assays

To measure adhesion, cells (50,000) were seeded in DMEM supplemented with 10% FBS. Adhered cells (15-30 min) were fixed, stained with crystal violet and counted using Eclipse TE300 Nikon microscope.

For clonogenicity assessment, cells (100 per 60 mm plates) were seeded in DMEM supplemented with 10%FBS, renewed every 3 days. After 4-11 days, colonies were stained with 0.005% crystal violet and counted.

### Western-blot analysis

Protein extracts and western blot analysis were performed as described 23 using either Anderson or SDS-gels for the electrophoresis. Membranes were probed with primary antibodies at 1:1000 dilution or as indicated: C3G H-300 (SCBT, sc-15359), C3G Home-designed for N-terminal domain (Genosphere), Vimentin (BD#550513), E-cadherin (BD#610182), Tyr1234/1235 P-MET (CST#3126), Tyr542 P-SHP2 (CST#3751); P-Thr180/Y182-p38MAPK (CST#9211), P-Thr202/Y204-ERKs (CST#9101), P-Ser473-Akt (CST#9271), Akt (CST#9272S), ERKs (CST#9102), p38 C-20 (SCBT, sc-535), P-Smad2 (CST#3101), Smad2/3 (CST#5678), Cytokeratin 19 (TromaIII, Hybridoma bank), β-actin (CST#3700, 1:2500) and α-Tubulin (CST#3873S, 1:2500).

### RNA extraction and RT-qPCR analysis

Total RNA was isolated using NucleoSpin RNA kit (Macherey-Nagel#740955.50) and reverse transcribed using SuperScript III-RT kit (Invitrogen). cDNA was amplified with specific primers for *Snail1, Zeb1, Zeb2, Twist1, Albumin, HNF4α, Alfa-fetoprotein* (*Afp*) using *Gusb* to normalize (Table 1) and detected by SYBR Green (Roche 04913850001) using 7900 Fast Real Time System (Life Technologies 4329001). Ct (threshold cycle) for a gene minus Ct for *Gusb*=ΔCt and then, referred to non-silenced control values (sample ΔCt-non-silenced ΔCt= ΔΔCt) to calculate RQ (2^-ΔΔCt^).

### Immunofluorescence analysis

Cells were seeded on glass coverslips pre-coated with 2% gelatin (Sigma#G9391) and maintained 24h in DMEM supplemented with 10% FBS. Cells were washed with PBS twice, fixed with 4% PFA (20 min) or cold methanol (2 min) for E-cadherin, and washed again with PBS. Then, cells were incubated with blocking solution (3% BSA-1.5% goat serum in PBS) for 1h at RT. Next, cells were incubated with anti-C3G (Home-designed, Genosphere), anti-E-cadherin (BD#610181), anti-Vimentin (CST#5741) or anti-ZO-1 (Invitrogen#33-9100) antibodies (1:50) in blocking solution, overnight at 4ºC. Then, cells were washed with PBS and incubated with secondary antibodies (1:200), goat anti-mouse-Alexa Fluor Plus 555 (Invitrogen#A32727) or goat anti-rabbit-Alexa Fluor Plus 555 (Invitrogen#A32732), and DAPI (1:1000) (Panreac-A4099) in blocking solution. After washing with PBS, coverslips were mounted with Prolong Gold Antifade Reagent (Invitrogen#P36930). Images were captured using a Nikon Eclipse TE300 microscope coupled to a camera or a laser confocal microscope (SP-8 Leica).

### Analysis of F-actin organization

Cells seeded on 2% gelatin-coated glass coverslips were fixed with 4% PFA for 20 min. F-actin was stained with rhodamine-conjugated phalloidin (Sigma Aldrich#P1951) as described 26. Images were analyzed by confocal microscopy.

### Analysis of cell surface MET

Serum-starved cells treated with HGF (20 ng/mL) for 10 min or 2 h or maintained untreated were detached with PBS-2 mM EDTA and incubated with blocking solution (1% BSA-PBS) for 15 min at 4ºC, followed by incubation with MET-Phycoerytrin (PE) antibody (5 µg/mL, Invitrogen#12-8854-80) or rat isotype control (5 µg/mL, Invitrogen#12-4001-81) in 0.1% BSA-PBS for 30 min at 4ºC. Fluorescence intensity was analyzed using a FACS Calibur flow cytometer.

### Cytometry analysis of CD44 and EpCAM

Cells were detached with trypsin-EDTA. For EpCAM detection, cells were incubated with anti-EpCAM-PE antibody (1:50, SCBT#66020) or control mouse isotype IgG (1:50, SCBT#2866) for 30 min at 4°C in 3% BSA-PBS. For CD44 analysis, cells were incubated with CD44-A488 antibody (1:50, Biolegend#103016) or control rat isotype IgG (1:50, Biolegend#400625) in 0.1% BSA-PBS. Fluorescence intensity was analyzed using a FACSCalibur flow cytometer.

### Induction of oval cell differentiation into hepatocytes

To induce differentiation into hepatocytes, 100,000 cells seeded on 60 mm plates pre-coated with collagen (Roche#11179179001) were maintained 24h in DMEM supplemented with 10% FBS. Next, medium was replaced by DMEM supplemented with 10% FBS, HGF (20 ng/mL), dexamethasone (1 µM, Sigma#D4902) and oncostatin M (10 ng/mL, R&D#495-MO). Cells were maintained for 3-9 days.

### Statistical analysis

Data are represented as the mean values ± S.E.M (n≥3) of independent experiments. Unpaired Student's t-test was used for comparison of two experimental groups and one-way or two-way ANOVA analyses to compare more than two groups with one or two variables using GraphPad Prism 7.0 software. Differences were considered significant when p value was p≤0.05.

## Results

### C3G knock-down enhances the migratory capacity of oval cells while reducing adhesion

**Figure 1:**
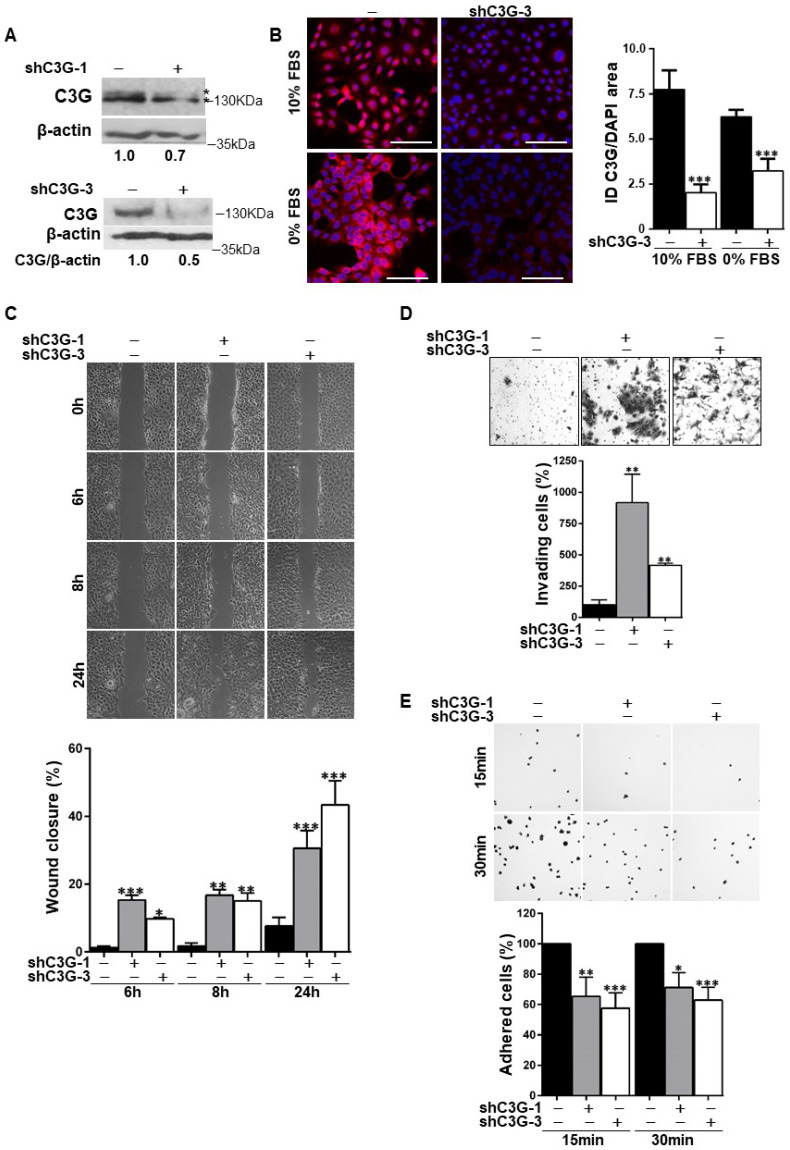
** C3G knock-down enhances migration and invasion in oval cells, while decreases adhesion.** Oval cells with permanent C3G knock-down using different shRNAs (shC3G-1 and shC3G-3) and non-silenced controls (-) were used. **A)** Western-blot analysis of C3G protein levels normalized with β-actin in cells maintained in the presence of serum. Different C3G isoforms are detected as a double band. **B)** C3G immunofluorescence (red) in cells maintained with either 10% FBS or 0% FBS for 16h as indicated. The histogram shows the mean value ± S.E.M of integrated intensity (ID) per DAPI area (n=4). Images were taken at the same exposure time. **C)** Wound-healing assay. Top panels, phase contrast microscopy images from cells at 0, 6, 8 and 24h after wound generation. Cells were maintained in the absence of serum. Lower panel, histogram showing the mean value ± S.E.M. of wound closure (n=4). **D)** Invasion through Matrigel using serum as chemoattractant. Top panel, representative images from invading cells. Lower panel, histogram showing the mean value ± S.E.M. of the number of invading cells (n=3-6). **E)** Adhesion assay. Top panel, representative images of adhered cells at 15 and 30 min after seeding in a medium supplemented with 10% FBS. Lower panel, histogram showing the mean value ± S.E.M. of adhered cells (percentage) (n=4-11). *p≤0.05, **p≤0.01 and ***p≤0.001, C3G-knock-down versus non-silenced cells.

We have previously described that oval cells express higher levels of C3G than adult hepatocytes 23. To evaluate the functional relevance of C3G in these cells we generated oval cells with permanent C3G knock-down using different shRNAs. A reduction in C3G protein levels upon shRNA-mediated knock-down was confirmed by western-blot and immunofluorescence analysis (Fig. 1A and 1B, and Supplementary Fig. 1A).

Considering that migration is necessary for the expansion of oval cells to repair the chronically damaged liver, and that C3G regulates cell adhesion, migration and invasion 21, 23, 26, we evaluated the effect of C3G down-regulation on these cellular processes. The analysis of cell migration using wound healing assays (Fig. 1C) revealed that wound closure in C3G-silenced cells was significantly higher at all analyzed time points (6, 8 and 24h). In agreement with the enhanced motility of C3G-silenced oval cells, invasion through Matrigel using serum as chemoattractant was also increased (Fig. 1D). As a reference, we used oval cells chronically treated with TGF-β, a well-characterized model of epithelial to mesenchymal transition (EMT) 8. The increase in invasion elicited by C3G down-regulation was similar to that induced by TFG-β in non-silenced cells (Supplementary Fig. 1B), but TGF-β further enhanced the invasive capacity of C3G-silenced cells (Supplementary Fig. 1B). Moreover, C3G knock-down oval cells showed decreased adhesion (Fig. 1E).

Therefore, oval cells with low levels of C3G show a higher migratory and invasive capacity and a lower adhesion, all of which could favor oval cell expansion.

### C3G down-regulation in oval cells promotes an EMT associated process with increased stemness markers

**Figure 2:**
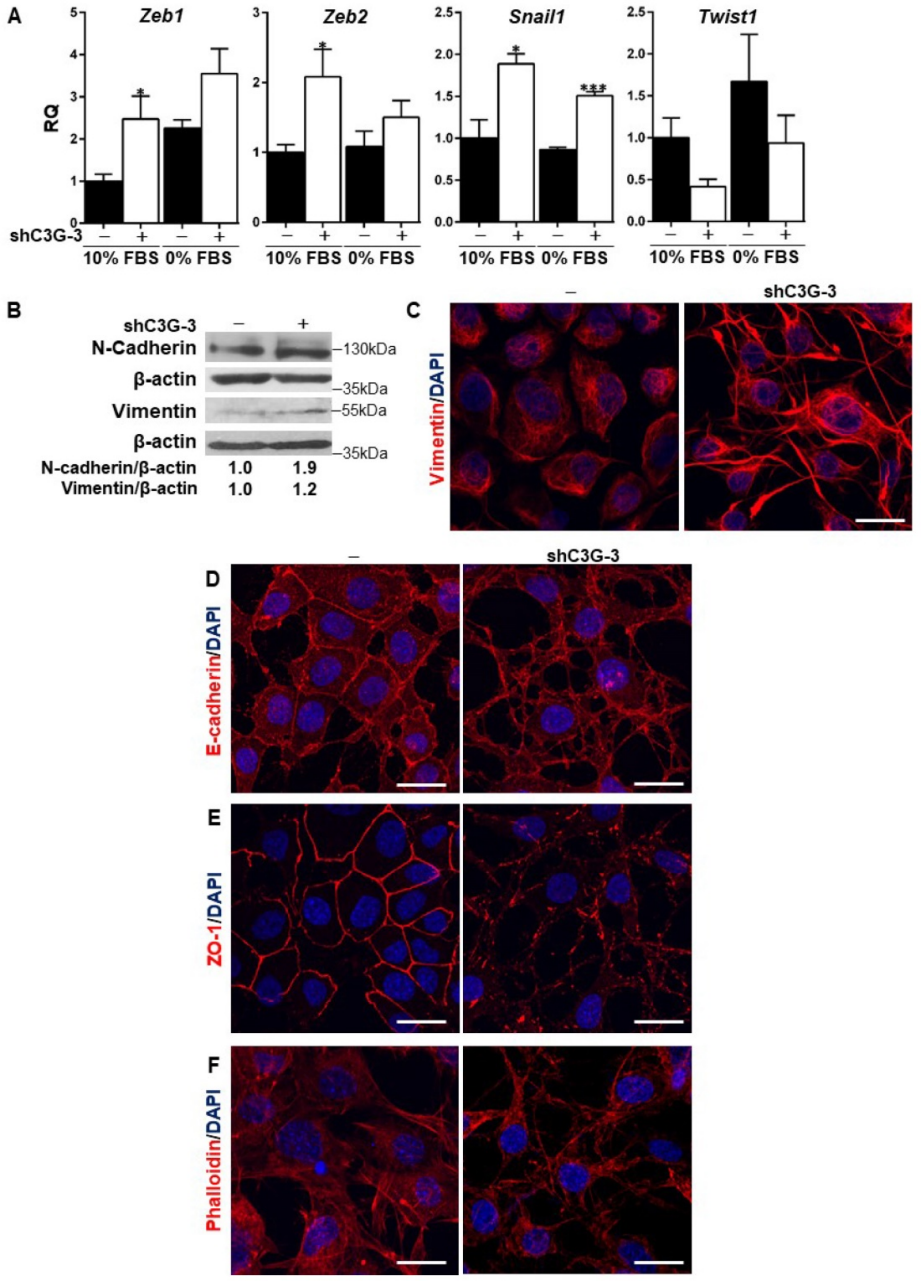
** C3G down-regulation promotes EMT in oval cells.** C3G knock-down (shC3G-3) oval cells and its non-silenced control (-) were used. **A)** Levels of *Snail1*, *Zeb1*, *Zeb2* and *Twist1* mRNAs in cells maintained either with 10%FBS or 0% FBS as indicated. Histograms show RQ mean value ± S.E.M. (n=4). *p≤0.05, and ***p≤0.001, C3G-knock-down versus non-silenced cells. **B)** Western-blot analysis of N-Cadherin and Vimentin protein levels normalized with β-actin. Cells were maintained with 10% FBS for 24h. Confocal microscopy images of Vimentin (**C**), E-Cadherin (**D**), ZO-1 (**E**) and F-actin (**F**) staining in cells maintained with 10% FBS. Nuclei were visualized with DAPI (blue). Images were taken at the same exposure time. Scale bars: 20 μm.

Previous data from the literature have demonstrated the relevance of TGF-β-induced EMT 8 for oval cell pro-regenerative capacity upon liver damage. C3G down-regulation is known to promote the acquisition of a more mesenchymal phenotype in HCC and glioblastoma cells 23, 26. Hence, we analyzed the potential induction of an EMT process in oval cells by C3G knock-down as a mechanism to enhance migration and invasion. Figure 2A shows increased mRNA levels of EMT-inducing transcription factors, *Snail1*, *Zeb1* and *Zeb2,* in C3G-silenced oval cells, when maintained both in the presence or absence of serum. Interestingly, this increase was not observed in *Twist1*, which showed a tendency to decrease. The increased expression of these transcription factors elicited by C3G down-regulation was similar to that induced by TGF-β treatment 8. Consistent with the increase in EMT-inducing transcription factors, the levels of the mesenchymal proteins, N-cadherin and Vimentin, were also higher in C3G-knock-down oval cells (Fig. 2B and 2C). This was corroborated upon C3G silencing with additional shRNAs (Supplementary Fig. 1A). Moreover, immunofluorescence analysis by confocal microscopy showed that Vimentin was differently distributed within C3G-silenced oval cells, being mainly present in cell extensions (Fig. 2C and Supplementary Fig. 1C). Confocal analysis also revealed the internalization and disorganized distribution of the epithelial marker, E-cadherin, in C3G knock-down cells, both when they were maintained in the presence or absence of serum (Fig. 2D and Supplementary Fig. 1D, respectively). The subcellular localization of the tight junction protein zona occludens-1 (ZO-1) also changed from a cell-to-cell membrane contact site distribution to a diffuse pattern within the membrane in C3G-silenced cells (Fig. 2E and Supplementary Fig. 1E). Lastly, F-actin staining revealed its accumulation at focal adhesions and cell extensions in C3G-knock-down oval cells, consistent with features of migratory cells, especially when maintained in the absence of serum (Fig. 2F and Supplementary Fig. 1F). All these data support the induction of an EMT process in oval cells by C3G silencing.

**Figure 3:**
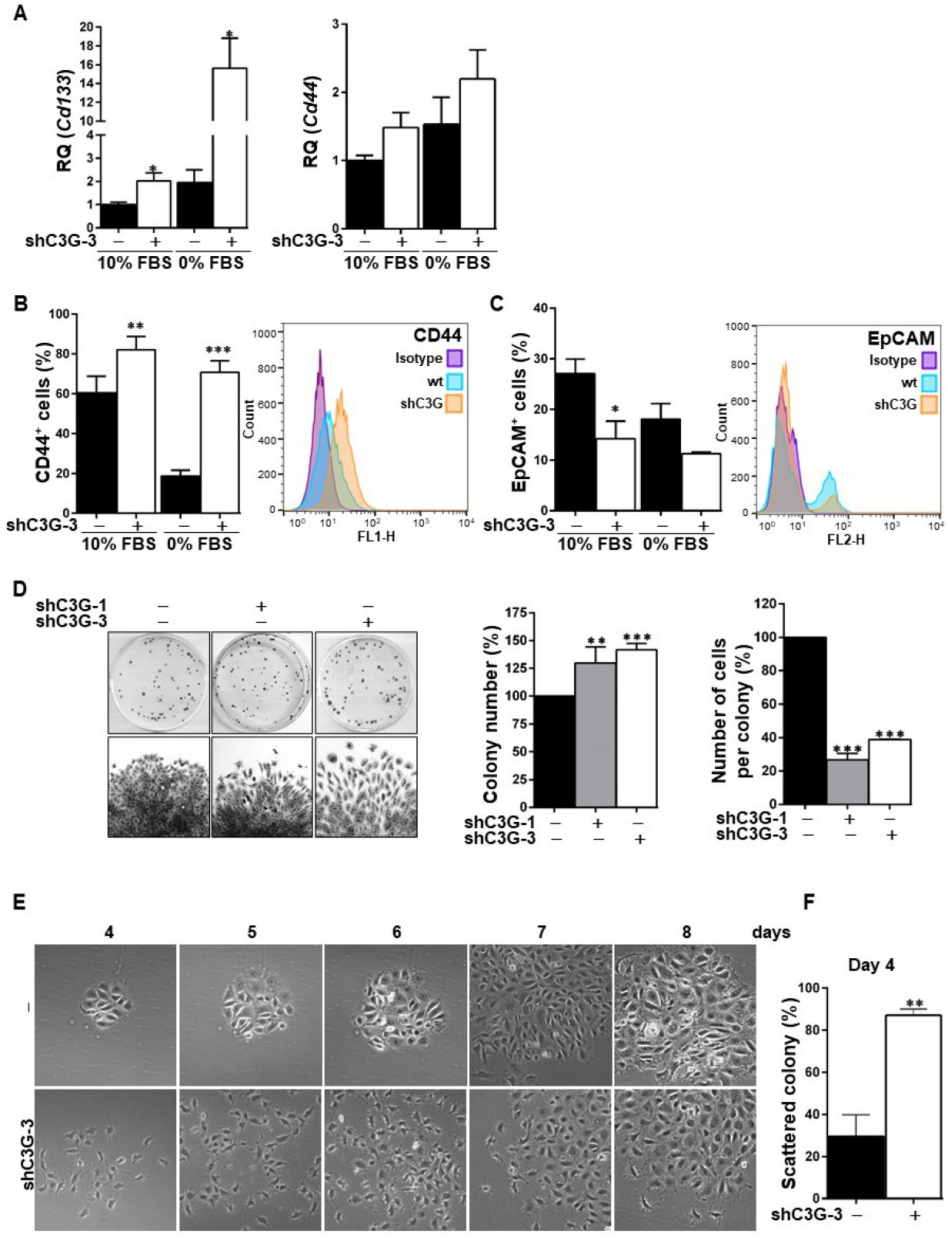
** C3G knock-down enhances oval cell stemness.** Oval cells with permanent C3G knock-down using different shRNAs (shC3G-1 and shC3G-3) and its non-silenced controls (-) maintained either with 10% FBS or 0% FBS. **A)** *Cd133* and *Cd44* mRNA levels. Histograms show RQ mean value ± S.E.M. (n=4). Cytometry analysis of CD44 **(B)** and EpCAM **(C)**. Left panels, histograms showing percentage of positive cells (mean value ± S.E.M.) (n=4). Right panels, fluorescence intensity. **D)** Clonogenic assay. Left panel, macroscopic view of colonies at day 11 (upper images), microscopic view of an individual colony (lower images). Right panels, histograms showing the mean value ± S.E.M. of the number of colonies or cells per colony (n=3-10). **E)** Microscopic view of individual colonies at days 4-8. **F)** Histograms showing mean value ± S.E.M. of scattered colonies (percentage) at day 4 (n=3). *p≤0.05, **p≤0.01 and ***p≤0.001, C3G-knock-down versus non-silenced cells.

The EMT process is often associated with the acquisition of stemness properties and markers 27, 28. To analyze whether C3G down-regulation could have an impact on the expression of hepatic stem/progenitor cell markers, *Cd133* and *Cd44* mRNAs levels were quantified by RT-qPCR (Fig. 3A). *Cd133* mRNA expression was significantly increased in C3G-knock-down cells, both in the presence and absence of serum. Likewise, *Cd44* mRNA levels were higher in C3G-silenced cells, although differences did not reach statistical significance. Moreover, flow cytometry analysis revealed that C3G down-regulation increased the percentage of CD44 positive cells and CD44 levels (Fig. 3B), while decreasing the percentage of cells expressing the epithelial marker EpCAM (Fig. 3C). These data indicate that the EMT process promoted in oval cells by C3G down-regulation is associated with enhanced expression of stemness-related markers.

Based on the need of a high clonal growth capacity for liver repair by oval cells, this was analyzed under adhesion conditions. As shown in Figure 3D, shC3G cells generated a significant higher number of colonies, reflecting a clonal growth advantage. However, the number of cells per colony was markedly reduced in shC3G cells. This could be explained by the enhanced pro-migratory capacity of these cells, which would facilitate cell escape from the original colony, leading to secondary colonies. This is supported by the morphological appearance of shC3G cells colonies, showing a pattern of scattered cells with less cell-to-cell contacts (Fig. 3E). This was already detected at early stages (day 4), when the number of scattered colonies was significantly increased in shC3G cells (Fig. 3F), and continued up to day 8, when cells were in closer proximity, but rarely in tight contact. However, it is important to highlight that C3G down-regulation did not promote anchorage-independent growth of oval cells (data not shown) and therefore, it did not confer tumorigenic capacity.

**Figure 4:**
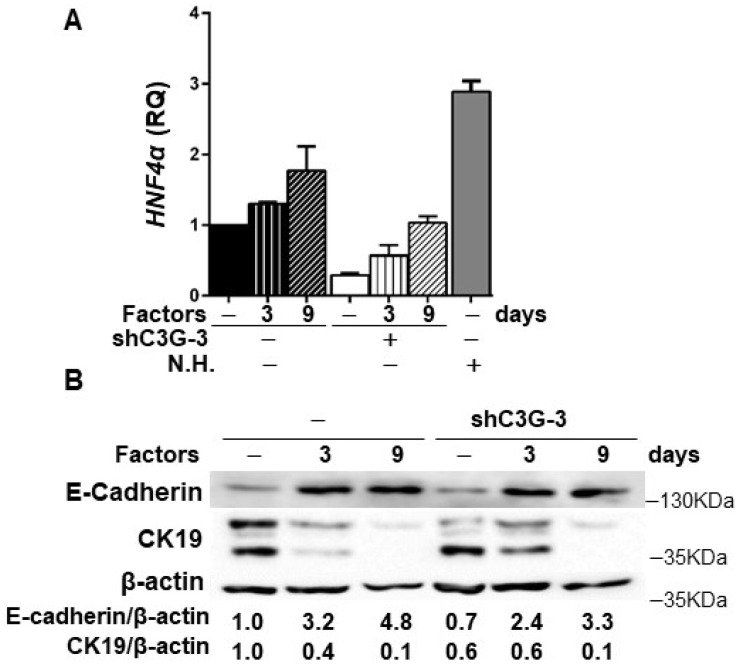
** Effect of C3G down-regulation on the differentiation of oval cells to hepatocytes.** C3G knock-down (shC3G-3) oval cells and its non-silenced control (-) were treated with HGF, dexamethasone and oncostatin M in a medium supplemented with 10% FBS for 3-9 days. N.H. (Neonatal hepatocytes). **A)** *HNF4α* mRNA levels. Histogram shows RQ mean value ± S.E.M. (n=2). **B)** Western-blot analysis of E-Cadherin and CK19 protein levels normalized with β-actin at time 0 (-) and at day 3 and 9 (n=3).

In addition, in agreement with the higher stemness of shC3G cells, changes in the expression of lineage markers were found, specifically, decreased mRNA levels of *Albumin* and *CK19* (Supplementary Fig. 2), a hepatocyte and cholangiocyte marker, respectively, widely used as oval cell markers; and reduced mRNA levels of the transcription factor *HNF4α*, a known driver of hepatocyte differentiation (Fig. 4A). No changes were found on the expression of *Afp*, an early hepatocyte differentiation marker (Supplementary Fig.2). Altogether, these data support that C3G down-regulation helps to maintain a non-differentiated phenotype in oval cells. Nevertheless, when cells were forced to differentiate into hepatocytes *in vitro* by treatment with a growth factor/hormone cocktail, a similar increase in the protein levels of E-cadherin and a decrease in CK19, were found in both parental and C3G-silenced cells (Fig. 4B). *HNF4α* mRNA expression increased in both control and shC3G oval cells, although it remained lower in shC3G cells at all the time points (Fig. 4A). All this suggests that either C3G is not essential for hepatocyte lineage commitment in oval cells or that low levels of C3G are sufficient for it.

In summary, low levels of C3G enhances pro-migratory, stemness and clonogenic properties without impairing differentiation into hepatocytes.

### C3G is required for HGF/MET signaling and functionality in oval cells and to maintain a proper TGF-β pathway activation

As mentioned previously, HGF/MET axis plays a key role in oval cells, enhancing survival and migration 6, 7, contributing to counterbalance TGF-β-induced EMT, and to maintain and/or promote their epithelial properties 8. On the other hand, we previously demonstrated that C3G is required for a full activation of MET signaling in HCC cells 23. Therefore, we analyzed whether C3G down-regulation had any effect on MET signaling activation and HGF/MET-induced invasion in oval cells.

**Figure 5:**
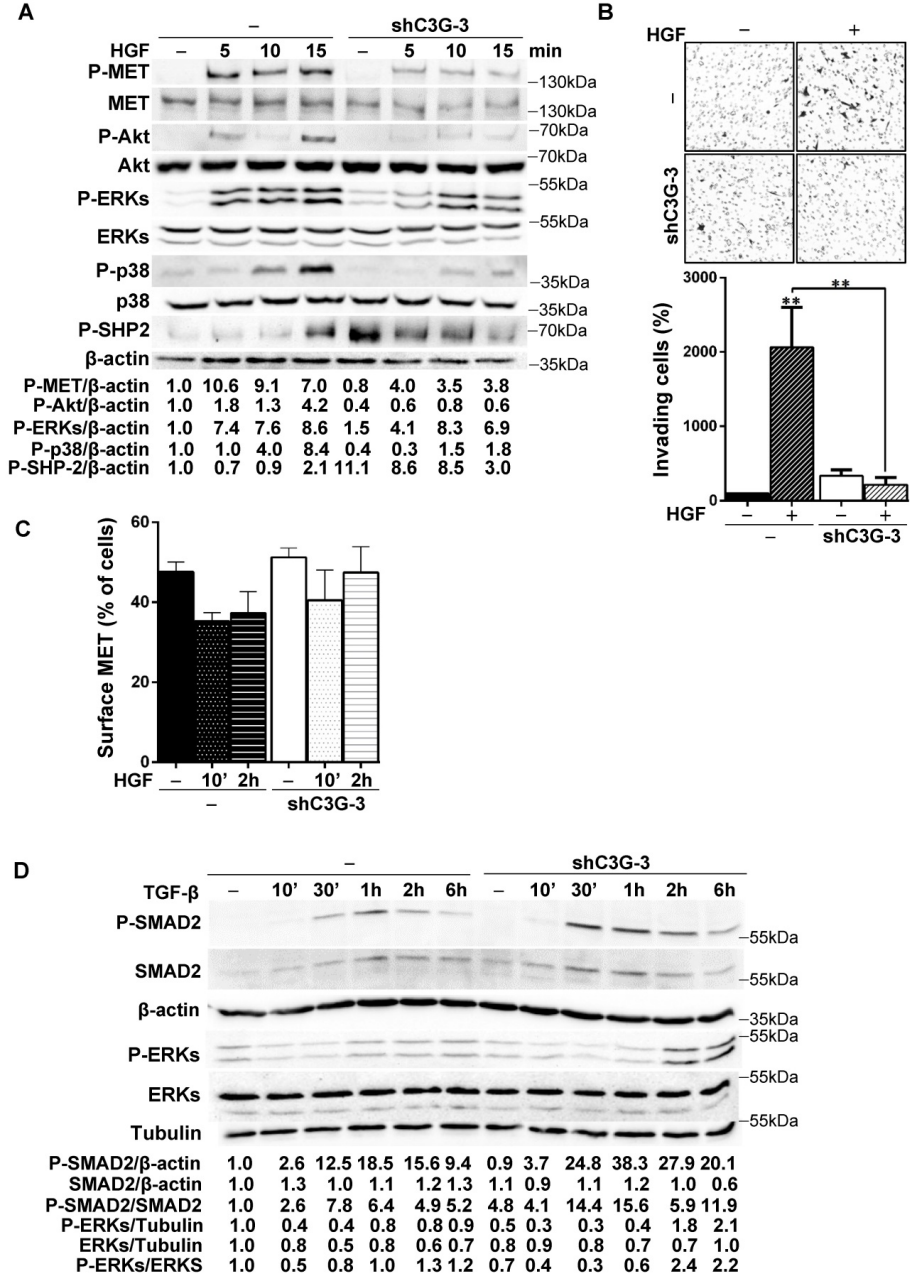
** C3G knock-down impairs HGF/MET signaling, while enhances the TGF-β one.** C3G knock-down (shC3G-3) oval cells and its non-silenced control (-) were used. **A)** Time-course analysis of P-MET, P-Akt, P-SHP2, P-p38MAPK, P-ERKs and total Akt, p38 and ERKs levels by western-blot normalized with β-actin (n=3). Cells maintained in the absence of serum for 16h, were treated with HGF for the indicated periods. **B)** HGF-induced invasion through Matrigel without chemoattractant. Top panel, representative images from invading cells. Lower panel, histogram showing the mean value ± S.E.M. of invading cells (percentage) (n=3). **C)** Cytometry analysis of MET cell surface levels in untreated or HGF-treated cells (10 min or 2h) maintained in the absence of serum. Histogram shows the mean value ± S.E.M. of the percentage of MET positive cells (n=4). **D)** Time-course analysis of P-Smad2, total Smad2, P-ERKs and total ERKs levels by western-blot normalized with β-actin, Tubulin or the corresponding non-phosphorylated protein (n=2). Cells maintained in the absence of serum for 16h, were treated with TGF-β for the indicated time periods. *p≤0.05, **p≤0.01 and ***p≤0.001, C3G-knock-down versus non-silenced cells or as indicated.

Figure 5A shows that HGF-induced MET phosphorylation decreased in shC3G oval cells. The levels of P-Akt and P-p38 MAPK were also highly reduced in these cells and ERKs activation was delayed and diminished. Contrarily, phosphorylation of SHP2 phosphatase was much higher both in unstimulated and HGF-stimulated shC3G cells up to 10 min, although after 15 min P-SHP2 levels were higher in parental cells. These results indicate that HGF/MET signaling is defective in oval cells with low levels of C3G. Therefore, we evaluated its functional consequences. We found that shC3G oval cells presented a lower invasive capacity in response to HGF (Fig. 5B). Furthermore, lack of a functional MET receptor mimicked the low adhesion shown by C3G-silenced oval cells (Supplementary Fig. 3).

To determine the mechanism responsible for this reduced HGF/MET signaling in oval cells with C3G down-regulation, we search for a potential alteration in MET membrane localization and/or recycling as C3G facilitates recycling and membrane localization of EGFR in glioblastoma (GBM) cells 26. However, we did not find significant changes in the levels of MET in the surface of shC3G oval cells (Fig. 5C). There was a tendency to decrease MET surface levels in non-silenced cells in response to HGF, which was not so well appreciated in shC3G cells. However, this effect was not significant. Alteration in HGF/MET signaling upon C3G knock-down could lead to an imbalance in the signals regulating oval cells, facilitating TGF-β effects, as HGF/MET counteract its action in oval cells 8. Indeed, P-Smad2 and P-ERKs levels were increased in shC3G cells in response to TGF-β (Fig. 5D), suggesting that canonical and non-canonical TGF-β signaling is enhanced in oval cells with C3G down-regulation, favoring TGF-β actions.

## Discussion

Adult HPCs/oval cells can proliferate and differentiate into hepatocytes and bile duct cells in response to a chronic and severe liver damage. However, they could also be potentially implicated in the generation of fibrosis and/or HCC 3, 29, 30. Therefore, a tight control of oval cell activation and expansion is essential. Moreover, due to its regenerative potential, a deep knowledge of how oval cell fate and biology is regulated constitutes a relevant issue.

C3G is highly expressed in oval cells and neonatal hepatocytes 23, pointing to its relevance in the liver and, particularly, in oval cells. However, it remains unknown the function of C3G in the liver 31. In this work, we have uncovered C3G as a protein that controls oval cell fate and physiology. C3G down-regulation by stable shRNA-mediated knock-down enhanced oval cell migratory and invasive properties to a similar extent to chronic treatment with TGF-β through inducing an EMT process. This effect of C3G down-regulation resembles that observed in colon carcinoma, HCC and GBM cells 21, 23, 26 and agrees with the inhibition of invasion promoted by C3G in breast cancer cells 32. Moreover, this EMT was accompanied by a greater expression of the stemness markers, CD133 and CD44, in shC3G oval cells, which may facilitate the maintenance and expansion of HPCs/oval cells, in agreement with its enhanced clonogenic capacity. Interestingly, this differs from the TGF-β-induced EMT in oval cells 8, which is not associated to increased stemness, suggesting different mechanisms to promote EMT in oval cells. This is further supported by the additional increase in invasion induced by chronic TGF-β treatment in C3G-silenced oval cells.

It is important to mention that the EMT induced by C3G down-regulation is a partial EMT. Hence, although the epithelial marker E-cadherin is partially internalized, it still coexists with mesenchymal markers (e.g. Vimentin and N-cadherin). This coexpression of epithelial and mesenchymal markers has been previously observed in HPCs in different contexts 33, including after TGF-β-induced EMT 8. Moreover, this is in line with the mesenchymal-epithelial transitional phenotype of HPCs from human fetal liver 34 and it could represent a mechanism to enhance the plasticity of oval cells, contributing to liver repair. In addition, we could hypothesize that reduced levels of C3G in HPCs/oval cells might prevent HCC development based on our previous published data showing that C3G is upregulated in these tumors promoting their growth 23. However, this deserves further investigation.

On the other hand, it should be noticed that a reduced expression of C3G did not impair *in vitro* oval cell differentiation into hepatocytes in response to HGF, dexamethasone and oncostatin M in the presence of serum, even though HGF/MET signaling was defective. These results could partially contradict previous data showing that C3G promotes the differentiation of muscle cells 35 and megakaryocytes 20, and with the fact that C3G knock-out in mouse embryonic stem cells (ESCs) impairs lineage commitment, while enhancing self-renewal and clonogenicity 36. This potential discrepancy might be due to differences in the levels of C3G expression. Thus, low levels of C3G in oval cells could still allow hepatocyte differentiation, while a total absence would not. On the other hand, the enhanced stemness of C3G knock-down hepatic progenitor/oval cells would agree with the above mentioned increased self-renewal found in C3G knock-out ESCs 36.

Considering the above comments, in the *in vivo* liver context, where several growth factors and cytokines are produced in the HPCs niche such as HGF, TGF-β, EGF, TWEAK, IL-6 or TNF-α 3, it is likely that C3G expression could be finely tuned in oval cells to promote a successful liver repair. Thus, reduced expression of C3G could facilitate oval cell expansion, particularly after the proliferation peak, to enhance migration. In agreement with this idea, a time-dependent decrease in C3G protein levels occurred in livers from DDC-treated mice to induce oval cell expansion (Supplementary Fig. 4A). In this line, in response to other types of liver damage, the expression of its mRNA (*RapGEF1*) was also reduced in the liver. Hence, the analysis of data from public databases indicate that *RapGEF1* mRNA expression decreased in liver samples from non-alcoholic fatty liver disease (NAFLD) and non-alcoholic steatohepatitis (NASH) patients compared to healthy livers (Supplementary Fig. 4B). However, there are some discrepancies in other studies that could be due to the complexity of the liver, where several cell types are present, in addition to oval cells, which can express *RapGEF1* mRNA and protein. On the other hand, since HPCs gradually lose EpCAM expression along with its maturation into hepatocytes 37, 38 and EpCAM inhibits hepatocytic differentiation in human liver progenitors, the decreased EpCAM levels in C3G knock-down oval cells might facilitate its differentiation into hepatocytes 39, also preventing the development of liver fibrosis 40.

It should be also mentioned that the defective HGF/MET signaling and function found in C3G knock-down oval cells is not due to a failure in MET membrane localization, but rather a consequence of an altered formation of signaling complexes, as described in HCC cells 23.

In conclusion, according to our work, C3G plays a key role regulating oval cell phenotype and fate, facilitating a balance in the signaling induced by different signals. Reduced C3G levels enhance the migratory and stemness capacity of oval cells, which might facilitate liver repair in response to chronic injury. Future studies will aim to further understand and characterize the mechanisms of C3G actions in oval cells and how C3G levels are regulated in the context of liver regeneration upon chronic damage.

## Supplementary Material

Supplementary figures.

## Figures and Tables

**Table 1:** Specific primers used for RT-qPCR analysis

Protein	Gene	Forward primer (5'-3')	Reverse primer (5'-3')
Snail1	*Snai1*	TCCAAACCCACTCGGATGTGAAGA	TTGGTGCTTGTGGAGCAAGGACAT
Zeb1	*Zeb1*	GTACAAACACCACCTGAAAGAGC	CCATTCACAGGCATCAAGC
Zeb2	*Zeb2*	AAAGCGTTCAAACACAAACACC	CCGCTTGCAGTAGGAGTACC
Twist	*Twist1*	CCGGAGACCTAGATGTCATTGT	CCACGCCCTGATTCTTGTGA
Albumin	*Alb*	ATCTGCACACTTCCAGAGAAG	TCCATGACAGTCTTCAGTTGC
HNF4*α*	*Hnf4α*	GGCATGGATATGGCCGACTAC	TTCAGATGGGGACGTGTCATT
AFP	*Afp*	TGTTGCCAAGGAAACTCG	GCAGCACTCTGCTATTTTGC
CD133	*Prom1*	CTGGGATTGTTGGCCCTCTC	AGGGCAATCTCCTTGGAATCA
CD44	*Cd44*	GGCCACCATTGCCTCAACTGT	TGCACTCGTTGTGGGCTCCTG
GUSB	*Gusb*	AAAATGGAGTGCGTGTTGGGTCG	CCACAGTCCGTCCAGCGCCTT
