# Supplementary material for: C3G down-regulation enhances pro-migratory and stemness properties of oval cells by promoting an epithelial-mesenchymal-like process
Source: Int J Biol Sci. 2022 Sep 25;18(15):5873–84. doi: 10.7150/ijbs.73192 (PMC9576514; doi:10.7150/ijbs.73192)
Supplement: Supplementary file 1 — Supplementary figures. [file ijbsv18p5873s1.pdf]

SUPPLEMENTARY INFORMATION

Supplementary figures

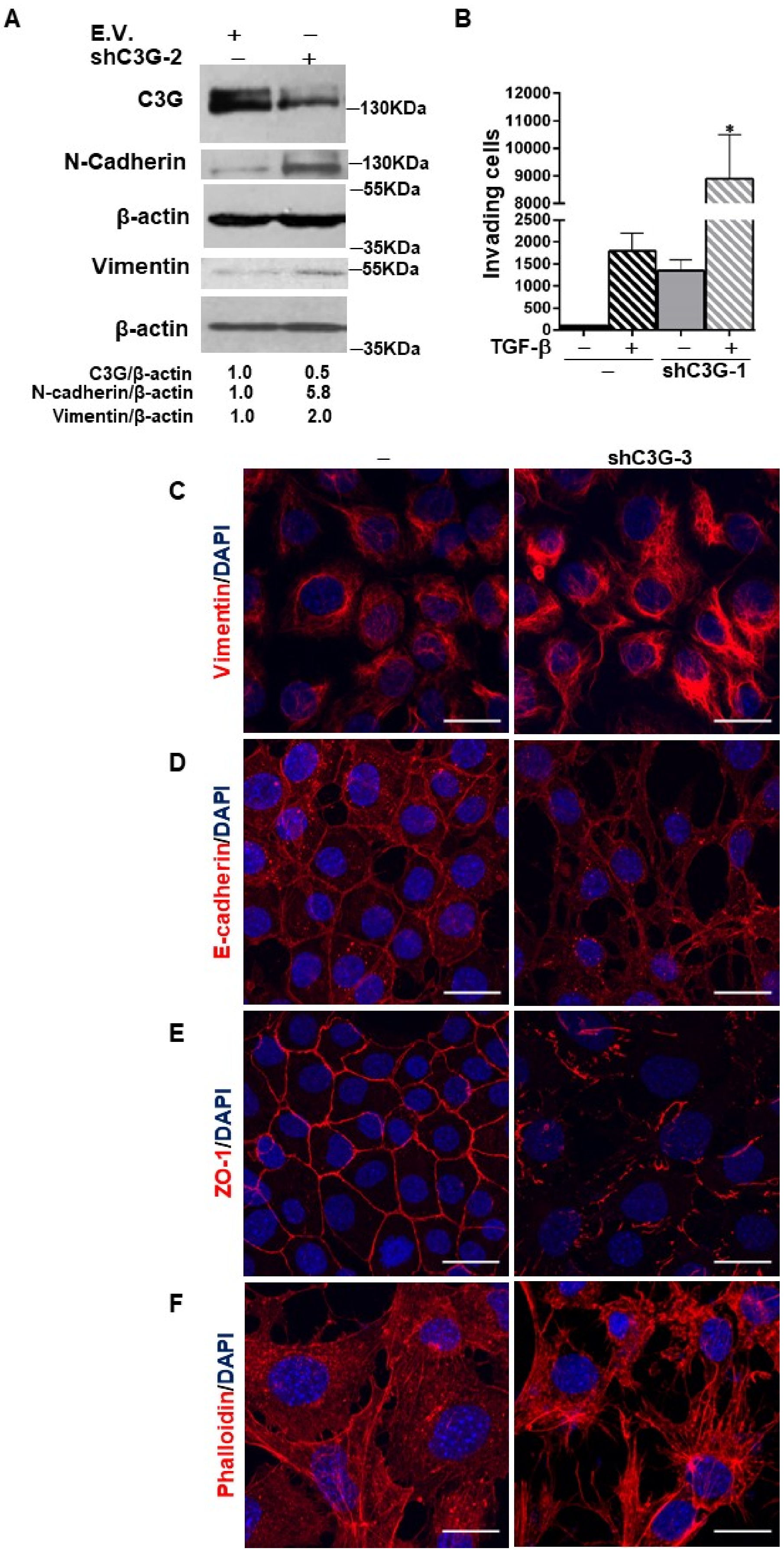

Supplementary figure 1-Chronic treatment with TGF-β mimicked the effect of C3G knock-down enhancing invasiveness of oval cells. Oval cells (C3G-silenced (shC3G-1, 2 or 3) and non-silenced (EV (transfected with the empty vector)) were maintained untreated in a medium supplemented with 10% FBS or chronically treated with TGF-β to induced EMT. **A)** Western-blot analysis of C3G, N-Cadherin and Vimentin protein levels normalized with β-Actin. **B)** Invasion assay through Matrigel using 10% FBS as chemoattractant. Histograms show the mean ± S.E.M. of number of invading cells (n=3). Confocal microscopy images of Vimentin (**C**), E-cadherin (**D**), ZO-1 (**E**) and Phalloidin F-actin (**F**) staining in cells maintained in the absence of serum. Scale bars: 20μm.

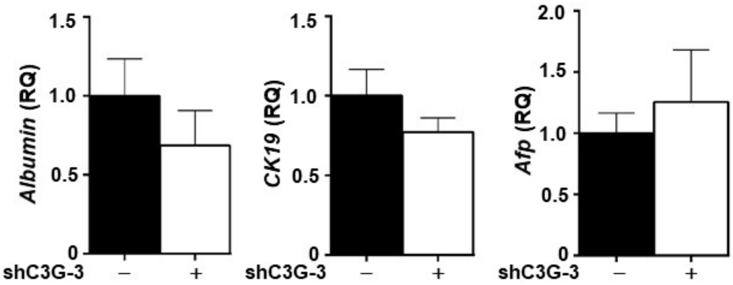

Supplementary figure 2-Effect of C3G knock-down on the expression of cytokeratin 19, albumin and alpha-fetoprotein mRNAs by oval cells. Levels of *CK19* (*KRT19*), *Albumin* and *Afp* mRNAs quantified by RT-qPCR in C3G-silenced (shC3G-3) and non-silenced oval cells. Histograms show RQ mean value ± S.E.M. (n=3).

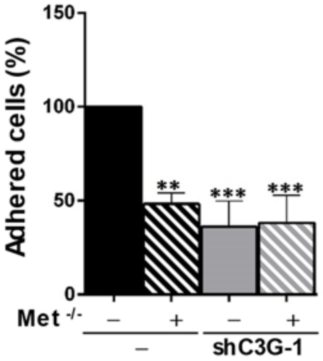

Supplementary figure 3-Lack of a functional MET receptor mimics the reduced adhesion of C3G knock-down in oval cells. Adhesion assay in oval cells with either C3G knock-down (shC3G) or lacking a functional MET receptor (Met-/-) (described in ref 5) and non-silenced wt cells. Histogram showing the mean value ± S.E.M. of the percentage of adhered cells at 15 min (n=3).

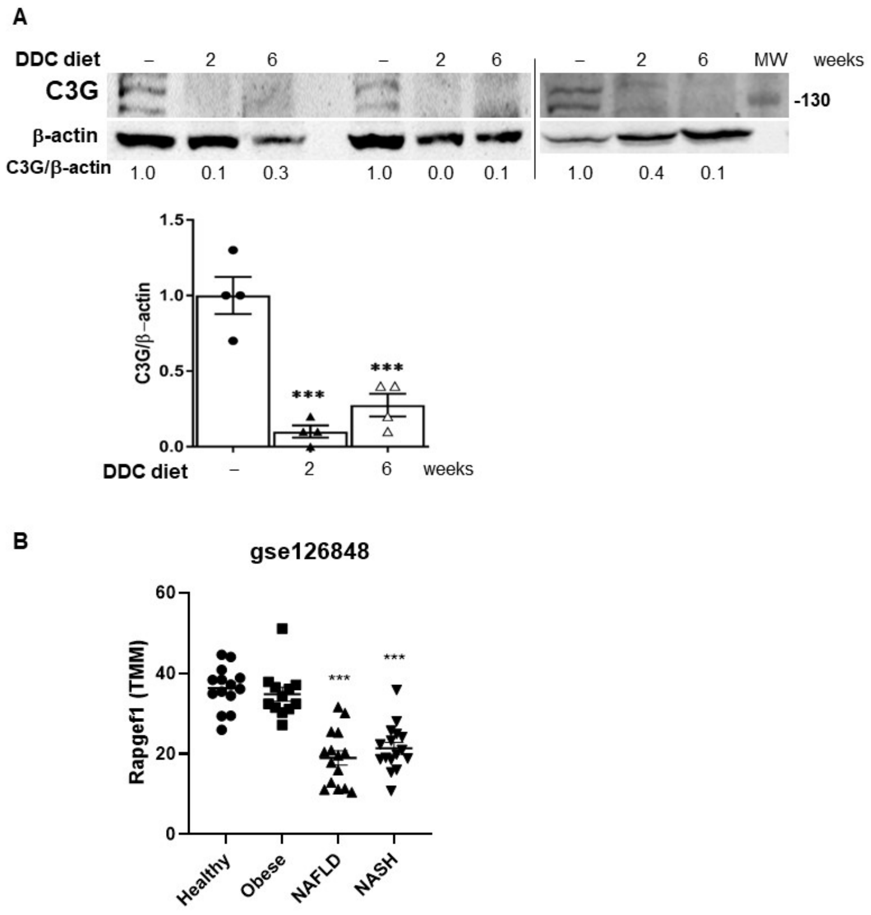

Supplementary figure 4-C3G expression is down-regulated in the liver upon chronic damage. (A) Western-blot analysis of C3G protein levels normalized with β-actin in liver samples from 3,5-Diethoxycarbonyl-1,4-Dihydrocollidine (DDC)-treated mice to induce oval cell expansion. Upper panel, images of representative western-blots showing different C3G isoforms with a line separating different blots; lower panel, histogram showing the quantification (mean value ± S.E.M.) of different blots referred to an untreated control, corresponding to 4 independent experiments. **(B)** Graphic showing *RapGEF1* mRNA expression in liver biopsies obtained from healthy normal weight (n=14) and obese (n=12) individuals, non-alcoholic fatty liver disease (NAFLD) with simple steatosis (n=15) and non-alcoholic steatohepatitis (NASH) (n=16) patients expressed as the median TMM (Trimmed Mean of M-values (counts per million normalized to TMM)). These data were extracted from NCBI GEO repository, accession number GSE 126848. ***p≤0.001 versus healthy samples.
